# Compatibility of Niuhuang Jiedu Tablets Results in Attenuated Arsenic Bioaccumulation and Consequent Protection against Realgar-Induced Toxicity in Mice

**DOI:** 10.1155/2022/7406694

**Published:** 2022-06-23

**Authors:** Wenfeng Xu, Shuo Xu, Yongmei Kuang, Xiaorong He, Pengfei Jin

**Affiliations:** ^1^Department of Pharmacy, Beijing Hospital, National Center of Gerontology, Institute of Geriatric Medicine, Chinese Academy of Medical Sciences, Beijing, China; ^2^Beijing Key Laboratory of Assessment of Clinical Drugs Risk and Individual Application (Beijing Hospital), Beijing 100730, China

## Abstract

Niuhuang Jiedu Tablets (NJT) is a popular over-the-counter traditional Chinese medicine (TCM) preparation. It is composed of realgar (As_2_S_2_) and seven other TCMs. The safety of NJT is of growing concern because arsenic (As) is carcinogenic to humans. The toxicity of realgar *in vivo* can mainly be attributed to the absorbed and accumulated As. This study investigated the correlation between the detoxification effects of the other TCMs in NJT on realgar and their influences on arsenic accumulation of realgar in mice. Histopathological examination, clinical biochemical test, and metabolic profiling analysis were used to evaluate the toxicity of realgar. The concentration of arsenic in mice whole blood as the hazard indicator was determined by inductively coupled plasma mass spectrometry (ICP-MS). The compatibility of NJT could decrease arsenic bioaccumulation of realgar in mice whole blood and consequently reduce the toxicity of realgar, which could be considered as one detoxification mechanism to realgar in NJT. The combination of *Rhei Radix et Rhizoma*, *Scutellariae Radix*, *Platycodonis Radix*, and *Glycyrrhizae Radix et Rhizoma* exhibited almost the same effects as NJT in regulating the serum biochemical parameters and metabolic profiles disturbed by realgar and in reducing arsenic accumulation of realgar in mice whole blood.

## 1. Introduction

Realgar (As_2_S_2_) is the sulphide mineral of arsenic (As) [1]. It has been used to treat external infectious dermatoses, convulsive epilepsy, malaria, and abdominal pain caused by parasitoses for hundreds of years. In recent years, realgar has also been shown to cure acute promyelocytic leukemia and other human malignancies effectively [2–4]. Traditional Chinese medicine (TCM) practitioners have accumulated rich experiences in combining arsenicals with other TCM materials. In the current edition of Chinese Pharmacopoeia (ChP), 1607 kinds of Chinese patent medicines are in use and 38 (2.4%) of them contain realgar, and some popular ones take realgar as the main component.

Niuhuang Jiedu Tablets (NJT) is one of the most popular over-the-counter TCM preparations with hundreds of years of clinical application history. According to ChP (2020), NJT is composed of realgar and seven other TCM materials, including Rengong Niuhuang (*Bovis Calculus Artifactus*), Bingpian (*Borneolum Syntheticum*), Shigao (*Gypsum Fibrosum*), Dahuang (*Rhei Radix et Rhizoma*), Huangqin (*Scutellariae Radix*), Jiegeng (*Platycodonis Radix*), and Gancao (*Glycyrrhizae Radix et Rhizoma*). It is a kind of antipyretic and detoxicate drug used for sore swollen throat, periodontitis, gingivitis, and mouth ulcer [5]. As is a widely distributed metalloid on the Earth. Epidemiological and clinical studies have indicated that As is associated with a variety of human cancers and noncancerous diseases. The Agency for Toxic Substances and Disease Registry (ATSDR) has placed As at the top of its Substance Priority List since 1997 [6]. Arsenic compounds have been identified as a carcinogenic hazard to humans by the International Agency for Research on Cancer (IARC) [7]. However, realgar is less toxic than soluble arsenicals and is even considered relatively safe depending on its median lethal dose. But exposure to realgar remains a toxicological risk and presents a safety concern of the public [8].

Realgar is poorly soluble in aqueous solutions, and it is an overestimate to evaluate the toxicity of realgar only by total arsenic content unless bioavailability is considered [9, 10]. The bioavailable portion of realgar can be absorbed and transported into blood. The absorbed arsenicals have a strong affinity with proteins containing thiol groups such as hemoglobin, metallothionein, and tubulin, so they accumulate with proteins in the form of stable complexes [11–13]. Therefore, compared with serum or plasma arsenic concentration, it is more reliable and scientific to assess the health risk of realgar and realgar-containing TCMs by whole blood arsenic concentration [11].

Realgar has been shown to cause acute or chronic toxic reactions in animals by some toxicological experiments [8, 14–17]. However, it became relatively safer after compatibility with NJT [11, 18–21]. In our previous study, the toxicity-attenuating effect of the compatibility of NJT with realgar was confirmed by metabonomics study [20, 21]. But the reason why the detoxification effect of each single TCM in NJT on realgar was not obvious puzzled us from then on. The attenuation of arsenic bioaccumulation of realgar by the synergistic effect of the other TCMs was considered to be one of the detoxification mechanisms [11, 18]. However, it remains unknown which of the other seven TCMs in NJT has this detoxification effect on realgar. The present study was designed to investigate the influences of the other TCMs in NJT on arsenic bioaccumulation of realgar and the relationship with their detoxification effects on realgar. Inductively coupled plasma mass spectrometry (ICP-MS) is a promising technique for trace element analysis in multiple research areas [22–24], and it has become a valuable tool for arsenic determination due to its low LOD, wide dynamic linear range, rapid detection, and few mass interferences [25–29]. In this experiment, whole blood arsenic accumulation of realgar was determined by ICP-MS. The toxicity of realgar was evaluated by histopathological examination, clinical biochemical test, and metabolic profiling analysis.

## 2. Materials and Methods

### 2.1. Materials and Reagents

Realgar was purchased from Xi'an Yuelai Pharmaceutical Technology Co., Ltd. (Xi'an, China). *Bovis Calculus Artifactus*, *Borneolum Syntheticum*, *Gypsum Fibrosum*, *Rhei Radix et Rhizoma*, *Scutellariae Radix*, *Platycodonis Radix*, and *Glycyrrhizae Radix et Rhizoma* were provided by Beijing Tongrentang Group Co., Ltd. (Beijing, China). Before the experiment, all TCMs were inspected and confirmed to meet the standards of ChP. The reference standard solution of arsenic (1000 mg/L) was purchased from the National Institute of Metrology (Beijing, China). Trace metal-grade nitric acid was supplied by Fisher Chemical (Fair Lawn, USA). 1 *μ*g/L tuning solution for ICP-MS with mixed elements (Ce, Co, Li, Mg, Tl, Y) and ICP-MS internal standard mix (100 mg/L of Bi, Ge, In, Li, Lu, Rh, Sc, Tb) were obtained from Agilent Technologies (Santa Clara, USA). Deuterium oxide (D_2_O, 99.8%) and sodium 3-trimethylsilyl [2, 2, 3, 3-^2^H_4_] propionate (TSP) were purchased from Alfa Aesar (Shanghai, China).

### 2.2. Animals and Treatments

Male Kunming mice with a weight of 25–35 g were obtained from SPF (Beijing) Biotechnology Co., Ltd. (Beijing, China). All animals were housed under controlled conditions (temperature 22–28°C, humidity 50–65%, and 12 h/12 h light-dark cycle). The animals were allowed to acclimate for 7 days with free access to food and water prior to the study. All animals were fasted overnight before the study. Animal studies were approved by the Institutional Animal Care and Use Committee (IACUC) of SPF (Beijing) Biotechnology Co., Ltd. and Scientific Research Department of Beijing Hospital (AEW2020010801).

Mice were randomly divided into 11 groups (12 mice/group), as shown in Table 1. All animals were subjected to oral administration for seven consecutive days. The dosage of realgar was set based on previous studies [11] and our preliminary experiments. NJT was prepared according to ChP. The weight ratio of realgar, *Bovis Calculus Artifactus*, *Borneolum Syntheticum*, *Gypsum Fibrosum*, *Rhei Radix et Rhizoma*, *Scutellariae Radix*, *Platycodonis Radix*, and *Glycyrrhizae Radix et Rhizoma* was 1 : 0.1 : 0.5 : 4: 4 : 3: 2 : 1 [5]. Blood samples were collected from the carotid artery of mice after they were anesthetized with ether on the 8th day. Six of the blood samples in each group were directly stored at −80°C until arsenic determination. The other six blood samples were centrifuged at 15000 × *g* for 10 min at 4°C to obtain serum samples, which were frozen at −80°C prior to analysis. Liver and kidney tissues were immediately removed, washed with 0.9% (w/v) NaCl solution, and soaked in 10% formalin solution.

### 2.3. Histopathology Analysis and Clinical Biochemistry

All liver and kidney tissues fixed in 10% formalin solution were embedded in paraffin wax and sectioned and stained with haematoxylin and eosin (H&E) for histopathological assessment. The measurement of aspartate aminotransferase (AST), alanine aminotransferase (ALT), alkaline phosphatase (ALP), blood urea nitrogen (BUN), and creatinine (CREA) in the serum was performed using a clinical analyzer (Hitachi, Ltd., Tokyo, Japan).

### 2.4. Pattern Recognition Analysis

Serum samples were thawed at room temperature and centrifuged at 15000 × *g* for 10 min. Then, 300 *μ*L of the supernatant was mixed with 100 *μ*L TSP (1 mg/mL) and 200 *μ*L D_2_O. The resulting solution was transferred into a 5 mm NMR tube. All NMR spectra were recorded on a Bruker AV 500 MHz spectrometer (Bruker GmbH, Germany) at 298 K. The Carr–Purcell–Meiboom–Gill (CPMG) sequence was employed to suppress the NMR signals from the macromolecules and allow the signals of micromolecules to be clearly presented. Sixty-four transients were collected into 64 K data points with the spectral width of 10 kHz. All free induction decays (FIDs) were zero-filled and multiplied by an exponential function with a 0.5 Hz line-broadening factor prior to Fourier transformation.

All spectra were manually phased, baseline adjusted, and referenced to TSP (*δ* 0.0) with MestReNova software (version 9.0.1, Mestrelab Research SL, Spain). The spectral region of *δ* 0.2–9.6 was integrated into segments with an equal spectral width of 0.04 ppm. The regions contributed to citrate (*δ* 2.70–2.64 and *δ* 2.58–2.52) were combined into two signals *δ* 2.66 and *δ* 2.54, respectively. The region at *δ* 4.6–5.2 was removed to eliminate baseline distortion caused by residual water resonance. The binned data were normalized to the total sum of spectral integrals to compensate for the concentration differences for all metabolites. Multivariate statistical analysis was conducted for the above normalized data using SIMCA-P software package (version 11.5, Umetrics AB, Sweden). Principal component analysis (PCA) was initially used to obtain a general overview of the metabolic pattern. Partial least square discriminant analysis (PLS-DA) was implemented on the mean-centered data to discover similarities or dissimilarities among the experimental groups. R^2^X, R^2^Y, and Q^2^ values can explain the quality of these models. R^2^X and R^2^Y indicate the robustness of the models, and Q^2^ represents the model predictability. The scores plots, in which each point represents an individual sample, were constructed to visualize the classification of the serum samples. The loadings plots, in which each point represents a single spectral region, were used to show NMR signals (metabolites) contributing to the clustering. Endogenous differential metabolites were subjected to MetaboAnalyst 4.0 (https://www.metaboanalyst.ca) to reveal disturbed biological pathways involved in the metabolism.

### 2.5. ICP-MS Analysis

0.5 mL of whole blood sample and 2 mL of trace metal-grade nitric acid were added to a microwave digestion tube. The digestion and preparation of the samples were carried out following our previously established method [30].

Total arsenic in blood was determined by an Agilent 7900 ICP-MS (Agilent Technologies International Japan, Ltd., Tokyo, Japan). MassHunter 4.5 Workstation Software was used for data acquisition. The equipment tuning was performed daily to assure responses of at least 3000 counts for Li, 10000 counts for Y, and 6000 counts for Tl and at most 2% for CeO/Ce and 3% for Ce^2+^/Ce. More operating details are given in Table 2. The samples and standard solutions were introduced into the ICP-MS nebulizer using a sample tube (1.02 mm, ID). The internal standard solution was obtained by diluting ICP-MS internal standard mix (Section 2.1) with 5% (v/v) HNO_3_ to give a concentration of 0.01 mg/L as Bi, Ge, In, Li, Lu, Rh, Sc, and Tb and injected online via an internal standard tube (0.19 mm, ID) throughout the data acquisition process.

Arsenic calibration standards were prepared by serially diluting the As standard solution with 5% HNO_3_, resulting in concentrations of 0.1, 1, 2.5, 5, 10, and 20 ng/mL. Calibration standards were freshly prepared each day. Quality control (QC) samples were prepared by spiking blank mice whole blood with As standards to reach final As concentrations of 5, 10, 400, and 600 ng/mL.

### 2.6. Statistical Analysis

All numerical data are expressed as mean ± SD. The data were statistically analyzed by one-way analysis of variance (one-way ANOVA) and Dunnett's *t*-test using SPSS 17.0 software (SPSS Inc., Chicago, USA). Values of *p* < 0.05 were considered statistically significant.

## 3. Results

### 3.1. Histopathology and Clinical Biochemistry

Histopathological examination of the liver and kidney samples showed no pathological changes among all the treated groups (Figures S1 and S2). The experimental dose of realgar did not possess the toxicity that could result in visible pathological changes. Serum biochemical parameters of all mice are displayed in Table 3. AST, ALT, and ALP levels increased significantly to varying degrees in groups R, RBC, RBS, and RGF compared to the control group. No significant difference in any biochemical parameter was observed among all the other experimental groups.

### 3.2. Metabolic Profiling Analysis

**Figure 1:**
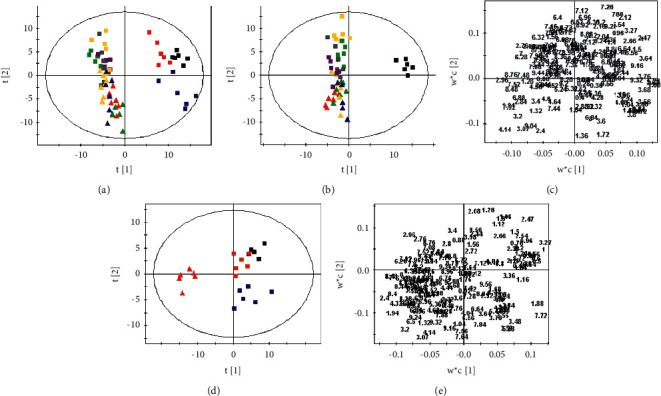
PCA scores plot (a) derived from ^1^H NMR spectra of the serum from the 11 groups (R^2^X = 0.834, *Q*^2^ = 0.706). PLS-DA scores plot and corresponding loadings plot based on ^1^H NMR spectra of the serum from the control group and groups R, RBC, RBS, RGF, RRR, RSR, RPR, RGR, and RFR (b, c) (R^2^X = 0.469, R^2^Y = 0.688, Q^2^ = 0.525) and from the control group and groups R, NJT, and RFH (d, e) (R^2^X = 0.503, R^2^Y = 0.735, *Q*^2^ = 0.604). Key: control group (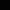), group R (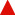), group NJT (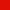), group RBC (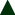), group RBS (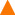), group RGF (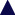), group RRR (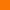), group RSR (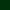), group RPR (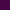), group RGR (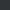), and group RFH (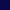).

The endogenous metabolites were characterized according to the literature and our previous studies [15, 17, 20, 21]. The metabolic profiles among control and experimental groups were further illustrated by pattern recognition analysis. The PCA scores plots based on ^1^H NMR spectra of the serum from the 11 groups are shown in Figure 1(a). It is observed that group R is separated distinctly from the control group. The points of groups RBC, RBS, RGF, RRR, RSR, RPR, and RGR cluster in the area of group R, while the points of groups NJT and RFH are distributed in the range of control group. The PLS-DA scores plot (Figures 1(b) and 1(d)) was used to sharpen the already observed separation. The corresponding loadings plot (Figures 1(c) and 1(e)) and one-way ANOVA were utilized for identification of potential differential metabolites that contributed to different metabolic profiles from comparative samples. The variable importance for projection (VIP) values of peak integral data in the loadings plot reflect the influences of the metabolites on the classification. Integral data with VIP values > 1 are considered to have above-average influence on the classification. One-way ANOVA was used to test the significance of differences in metabolites among groups, with *p* < 0.05 as the standard.

From the PLS-DA scores plot, the obvious separation of group R from the control group is observed (Figure 1(b)). Based on VIP values (VIP > 1.0) and *p* values (*p* < 0.05), the separation is attributed to the elevation in the levels of lactate (*δ* 1.32, d, and *δ* 4.14, q), acetate (*δ* 1.94, s), pyruvate (*δ* 2.40, s), creatine (*δ* 3.07, s), and choline (*δ* 3.20, s) with the reduction in the levels of leucine and isoleucine (*δ* 0.96–1.00, m), valine (*δ* 1.00, d, and *δ* 1.06, d), alanine (*δ* 1.50, d), 2-oxoglutarate (*δ* 2.47, m), citrate (*δ* 2.54, d, and *δ* 2.66, d), and trimethylamine-N-oxide (TMAO, *δ* 3.27, s) in group R compared with the control group. The metabolic profiles of groups RBC, RBS, RGF, RRR, RSR, RPR, and RGR were in line with that of group R. As shown in Figure 1(d), groups NJT and RFH could be easily distinguished from group R. According to the results of the loadings plot (Figure 1(e)) and statistical analysis, the changes in the concentrations of the above endogenous metabolites in group R were modulated to be close to the normal state in groups NJT and RFH.

**Figure 2:**
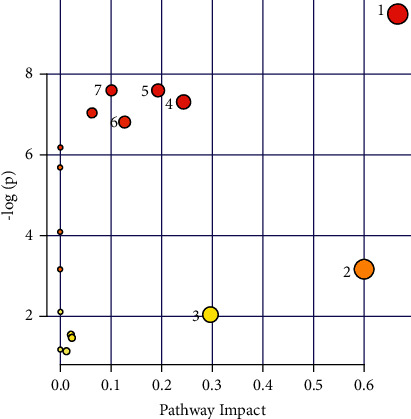
The summary of pathway analysis in the serum of realgar-exposed mice compared with control mice. Each circle represents one metabolic pathway; the size of circle shows the positive correlation with the impact of each pathway; the colour of circle denotes the significance from the highest value in red to the lowest value in white. (1) Valine, leucine, and isoleucine biosynthesis; (2) synthesis and degradation of ketone bodies; (3) glyoxylate and dicarboxylate metabolism; (4) pyruvate metabolism; (5) citrate cycle; (6) glycolysis or gluconeogenesis; (7) butanoate metabolism.

Metabolic profiling analysis provides a comprehensive and sensitive map of endogenous metabolites associated with toxic reactions to realgar. The biological pathways involved in the metabolism of the above-mentioned metabolites were determined by pathway analysis. The results are presented graphically (Figure 2) and in a detailed table (Table 4). The pathways with the impact value > 0.1 were considered closely related to realgar-induced toxicity [31]. Consequently, valine, leucine, and isoleucine biosynthesis, synthesis and degradation of ketone bodies, glyoxylate and dicarboxylate metabolism, pyruvate metabolism, citrate cycle, glycolysis or gluconeogenesis, and butanoate metabolism were recognized as the influenced metabolic pathways associated with the toxicity of realgar.

### 3.3. Method Validation Results

The method was validated in detail based on our previous experiment [30]. The calibration curve and linearity parameters over the range of 0.1–20 ng/mL for As was automatically generated with MassHunter 4.5 Workstation Software. The calibration curve was *Y* = 0.3185*X* + 0.0143 with a correlation coefficient (*r*) of 0.9999, and the LOQ was 0.043 ng/mL.

The precision and accuracy results are summarized in Table 5. Good precision and accuracy were acquired for As at the four different levels. The RSD values of intrabatch and interbatch precision were between 4.17% and 9.89% and 3.80% and 8.35%, respectively. The relative error (RE) of the four selected concentrations were −9.00%, −7.40%, −3.81%, and −1.32%, respectively. The recoveries which were investigated at each QC level were between 93.15% and 101.02%.

### 3.4. Determination of Whole Blood Arsenic Accumulation

**Figure 3:**
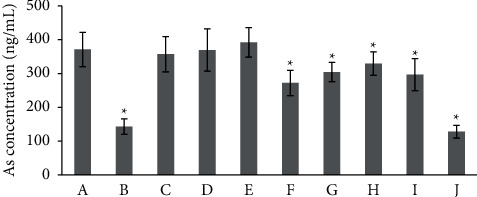
Arsenic accumulation in whole blood of mice. (A) Group R (B) Group NJT. (C) Group RBC. (D) Group RBS. (E) Group RGF. (F) Group RRR. (G) Group RSR. (H) Group RPR. (I) Group RGR. (J) Group RFH. ^*∗*^*p* < 0.05 versus group R (*n* = 6).

The total blood arsenic concentration of mice is shown in Figure 3. The whole blood arsenic concentrations of mice in groups RBC, RBS, and RGF were not significantly different from those of group R. The amounts of As in mice whole blood of groups RRR, RSR, RPR, and RGR did not increase as much as that of group R. The As accumulated in mice whole blood of group NJT decreased by about 60% compared with the realgar solo group. The result of group RFH was similar to that of group NJT, which indicated that *Rhei Radix et Rhizoma*, *Scutellariae Radix*, *Platycodonis Radix*, and *Glycyrrhizae Radix et Rhizoma* synergistically reduced the amount of arsenic accumulated in mice as the whole prescription.

## 4. Discussion

The dose of realgar used in this study did not cause pathological changes in the liver and kidney of mice. Significant increases in serum AST, ALT, and ALP levels were observed in groups R, RBC, RBS, and RGF, which proved that liver injury occurred. Although the changes were not statistically significant, the levels of AST, ALT, and ALP in groups RRR, RSR, RPR, and RGR were found to increase to different degrees compared with that of the control group. The elevated levels of AST, ALT, and ALP due to realgar injury could be decreased by NJT treatment, which suggested that NJT had hepatoprotective effects on realgar-induced liver injury. The combination of *Rhei Radix et Rhizoma*, *Scutellariae Radix*, *Platycodonis Radix*, and *Glycyrrhizae Radix et Rhizoma* exhibited the same effect as the whole prescription in regulating the serum biochemical parameters disturbed by realgar.

Conventional biological assessment of drug toxicity is based on targeted analysis of a small number of measured indicators, which provides limited opportunities to detect the trends and characterize mechanisms of toxicity [32]. The variations of endogenous metabolic networks reflect different pathological conditions and characterize the biochemical mechanisms of xenobiotics in humans or animals [33]. Metabolic profiling analysis makes recognition of the consistently varying endogenous metabolites and elucidation of changes in the associated metabolic regulatory networks come true [34]. As a convenient and effective tool, metabolic profiling analysis has been used to elucidate the toxicity of TCMs [35]. This study investigated the effects of the other TCMs in NJT on realgar-induced toxicity in mice. The metabolic profiles of mice influenced by realgar could not be altered by combining realgar with any other single TCM in NJT. However, the compatibility of them could reduce the toxicity of realgar. The concentrations of leucine, isoleucine, valine, lactate, acetate, alanine, pyruvate, 2-oxoglutarate, citrate, creatine, choline, and TMAO affected by realgar in mice were positively regulated toward normal levels by NJT. These metabolites were interrelated and affected many metabolic pathways. The combination of *Rhei Radix et Rhizoma*, *Scutellariae Radix*, *Platycodonis Radix*, and *Glycyrrhizae Radix et Rhizoma* could reduce the toxicity of realgar in mice as NJT. This finding with mouse as the animal model was consistent with our previous research using rats as the experimental animals [20]. From the perspective of correlation to the influence on the toxic sources of realgar, the possible reason why each single TCM in NJT did not show the toxicity alleviation effect on realgar and the possible underlying mechanism of their synergistic detoxification effect on realgar were also explored in the present study.

The toxicity of realgar can mainly be attributed to the absorbed and accumulated soluble As. Some herbal ingredients in TCM patent medicines containing toxic minerals showed detoxification effect by inhibiting the bioaccessibility or bioavailability of toxic elements [36, 37]. The bioaccessibility of soluble As in NJT was significantly lower than that of realgar in simulated gastrointestinal juice *in vitro* [38–40]. Furthermore, *in vivo* experiment revealed that NJT could significantly inhibit the arsenic absorption and reduce the concentration of total As in tissues compared to realgar [11, 18]. In this study, arsenic accumulation in whole blood of mice was measured to attempt to reveal the different toxic reactions of mice in each group to realgar. *Bovis Calculus Artifactus*, *Borneolum Syntheticum*, and *Gypsum Fibrosum* did not influence the arsenic accumulation and toxicity of realgar in mice, simultaneously. *Rhei Radix et Rhizoma*, *Scutellariae Radix*, *Platycodonis Radix*, and *Glycyrrhizae Radix et Rhizoma* could decrease the As accumulated in whole blood of mice to different degrees, respectively. However, the accumulated As in mice of these four groups could still result in toxic reactions as in group realgar. It was speculated that the amount of As accumulated in these four groups were higher than the normal tolerable amount of As in mice, which requires further study. The combination of *Rhei Radix et Rhizoma*, *Scutellariae Radix*, *Platycodonis Radix*, and *Glycyrrhizae Radix et Rhizoma* dramatically inhibited the arsenic accumulation of realgar in mice, which was consistent with the experimental result of group NJT. Meanwhile, the mice from groups RFH and NJT did not display the toxic responses as the mice from group realgar. The reduction of arsenic accumulation of realgar in mice by *Rhei Radix et Rhizoma*, *Scutellariae Radix*, *Platycodonis Radix*, and *Glycyrrhizae Radix et Rhizoma* in terms of decreasing absorption and increasing excretion of As will be investigated in our further studies, which are still ongoing and require more work.

## 5. Conclusion

In the present study, the correlation between the effects of the other TCMs in NJT on the toxicity of realgar and their influences on its arsenic accumulation was assessed *in vivo*. The toxicity of realgar was evaluated by histopathological examination, clinical biochemical test, and metabolic profiling analysis. The concentration of As accumulated in mice whole blood was determined by ICP-MS. NJT could decrease arsenic accumulation of realgar in whole blood of mice and consequently alleviate its toxicity. *Rhei Radix et Rhizoma*, *Scutellariae Radix*, *Platycodonis Radix*, and *Glycyrrhizae Radix et Rhizoma* were found to be the herbal basis of this detoxification effect on realgar in NJT.

## Figures and Tables

**Table 1:** Grouping scheme of this experiment.

	Group name										
	Control	R	NJT	RBC	RBS	RGF	RRR	RSR	RPR	RGR	RFH
Realgar (R, 0.3 g/kg)		√	√	√	√	√	√	√	√	√	√
*Bovis Calculus Artifactus* (BC, 0.03 g/kg)			√	√							
*Borneolum Syntheticum* (BS, 0.15 g/kg)			√		√						
*Gypsum Fibrosum* (GF, 1.2 g/kg)			√			√					
*Rhei Radix et Rhizoma* (RR, 1.2 g/kg)			√				√				√
*Scutellariae Radix* (SR, 0.9 g/kg)			√					√			√
*Platycodonis Radix* (PR, 0.6 g/kg)			√						√		√
*Glycyrrhizae Radix et Rhizoma* (GR, 0.3 g/kg)			√							√	√

Key: √, corresponding TCM included.

**Table 2:** ICP-MS operating parameters.

Parameter	Value
RF power	1550 W
RF matching	1.2 V
Sampling depth	10.0 mm
Nebulizer gas	1.07 L/min
Makeup gas	−
Nebulizer pump	0.1 rps
S/C temperature	2°C
Acquisition mode	Spectrum
Peak pattern	3 points
Replicates	3
Sweeps per replicate	100
Monitored elements for calculations	^75^ As and ^72^Ge
Integration time per mass	1.0 s for ^75^ As, 0.3 s for ^72^Ge
Uptake speed	0.3 rps
Uptake time	40 s
Stabilization time	40 s

**Table 3:** Clinical chemistry parameters.

Group name	Biochemical parameters				
	AST (U/L)	ALT (U/L)	ALP (U/L)	BUN (mmol/L)	CREA (*μ*mol/L)
Control group	85.92 ± 7.88	30.37 ± 5.58	98.12 ± 10.86	6.78 ± 0.74	45.38 ± 5.35
Group R	106.12 ± 12.39^*∗*^	43.68 ± 4.71^*∗*^	116.48 ± 16.17^*∗*^	7.47 ± 1.39	47.22 ± 7.58
Group NJT	79.15 ± 6.91	28.83 ± 3.30	90.43 ± 12.17	7.87 ± 1.56	48.47 ± 8.02
Group RBC	111.30 ± 14.14^*∗*^	45.52 ± 4.90^*∗*^	123.12 ± 15.87^*∗*^	8.43 ± 1.23	52.32 ± 7.33
Group RBS	103.42 ± 10.01^*∗*^	40.63 ± 6.20^*∗*^	116.12 ± 12.37^*∗*^	7.77 ± 2.12	45.92 ± 13.65
Group RGF	113.17 ± 13.15^*∗*^	48.73 ± 3.46^*∗*^	131.07 ± 12.21^*∗*^	7.03 ± 1.56	50.92 ± 8.65
Group RRR	90.92 ± 9.79	34.66 ± 3.52	101.78 ± 13.15	6.37 ± 0.95	52.12 ± 8.07
Group RSR	88.52 ± 5.84	36.07 ± 4.18^*∗*^	105.63 ± 15.89	6.18 ± 1.24	47.03 ± 8.52
Group RPR	93.87 ± 6.88	33.47 ± 4.06	112.63 ± 11.06	7.82 ± 1.89	43.18 ± 9.32
Group RGR	97.22 ± 9.00	35.52 ± 6.80	108.63 ± 14.92	8.17 ± 1.65	50.10 ± 8.03
Group RFH	90.80 ± 9.97	26.05 ± 7.39	99.18 ± 15.37	7.88 ± 1.64	42.47 ± 7.97

Statistics: ^*∗*^*p* < 0.05 versus the control group. Data are presented as mean ± SD of six animals per group.

**Table 4:** Results of the pathway analysis.

Pathway name	Compounds	Hits	Raw *p*	−Log (*p*)	Holm *p*	FDR	Impact
Valine, leucine, and isoleucine biosynthesis	11	3	0.000076	9.4822	0.0061718	0.0061718	0.66666
Butanoate metabolism	20	3	0.000504	7.593	0.040316	0.013607	0.10145
Citrate cycle (TCA cycle)	20	3	0.000504	7.593	0.040316	0.013607	0.19339
Pyruvate metabolism	22	3	0.000674	7.302	0.052587	0.013652	0.24337
Alanine, aspartate, and glutamate metabolism	24	3	0.000878	7.0384	0.067568	0.014216	0.06329
Glycolysis or gluconeogenesis	26	3	0.0011163	6.7977	0.084841	0.01507	0.12753
Glycine, serine, and threonine metabolism	32	3	0.0020683	6.181	0.15512	0.023934	0
Valine, leucine, and isoleucine degradation	38	3	0.0034165	5.6791	0.25282	0.034592	0
Aminoacyl-tRNA biosynthesis	67	3	0.016835	4.0843	1	0.15151	0
D-Glutamine and D-glutamate metabolism	5	1	0.042129	3.167	1	0.31022	0
Synthesis and degradation of ketone bodies	5	1	0.042129	3.167	1	0.31022	0.6
Selenoamino acid metabolism	15	1	0.12155	2.1075	1	0.7472	0
Pantothenate and CoA biosynthesis	15	1	0.12155	2.1075	1	0.7472	0
Glyoxylate and dicarboxylate metabolism	16	1	0.12915	2.0468	1	0.7472	0.2963
Cysteine and methionine metabolism	28	1	0.21577	1.5336	1	1	0.02103
Glycerophospholipid metabolism	30	1	0.22941	1.4722	1	1	0.02315
Tyrosine metabolism	42	1	0.3068	1.1815	1	1	0
Arginine and proline metabolism	44	1	0.31899	1.1426	1	1	0.01198

*Note.* Compounds represent the total number of compounds involved in the pathway; Hits represent the actually matched number from the user uploaded data; Raw *p* is the original *p* value calculated from the enrichment analysis; Holm *p* is the *p* value adjusted using the Holm–Bonferroni method; FDR is the *p* value adjusted by the false discovery rate; Impact is the pathway impact value calculated from pathway topology analysis [31].

**Table 5:** Precision, accuracy, and recovery of total arsenic in mice whole blood.

Theoretical concentration (ng/mL)	Found (ng/mL) (mean ± SD, *n* = 5)			Overall mean (ng/mL)	Intrabatch RSD (%) (*n* = 5)	Interbatch RSD (%) (*n* = 15)	RE (%) (*n* = 15)	Recovery (%) (mean ± SD, *n* = 5)
	Batch 1	Batch 2	Batch 3					
5	4.62 ± 0.46	4.66 ± 0.40	4.38 ± 0.27	4.55	9.89	8.35	−9.00	93.29 ± 5.46
10	9.41 ± 0.64	9.02 ± 0.73	9.34 ± 0.52	9.26	6.80	6.62	−7.40	94.56 ± 1.90
400	395.28 ± 22.13	375.12 ± 16.94	383.92 ± 22.53	384.77	5.60	5.45	−3.81	97.13 ± 2.70
600	575.20 ± 23.99	597.44 ± 13.99	603.68 ± 21.05	592.11	4.17	3.80	−1.32	98.34 ± 2.05

## Data Availability

The data used to support the findings of this study are available from the corresponding authors upon request.

## Supplementary Materials

Supplementary Materials
*Figure S1*. Photomicrographs of representative sections of the liver from the control group (a), group R (b), group NJT (c), group RBC (d), group RBS (e), group RGF (f), group RRR (g), group RSR (h), group RPR (i), group RGR (j), and group RFH (k). *Figure S2*. Photomicrographs of representative sections of the kidney from the control group (a), group R (b), group NJT (c), group RBC (d), group RBS (e), group RGF (f), group RRR (g), group RSR (h), group RPR (i), group RGR (j), and group RFH (k).

## References

[1] FuRuta E., Ishihara S., Okumura R., Iinuma Y. (2015). Analysis of toxic elements in Chinese medicines and herbs. *Journal of Radioanalytical and Nuclear Chemistry*.

[2] Ding W., Zhang L., Kim S. (2015). Arsenic sulfide as a potential anti-cancer drug. *Molecular Medicine Reports*.

[3] Liu J. X., Zhou G. B., Chen S. J., Chen Z. (2012). Arsenic compounds: revived ancient remedies in the fight against human malignancies. *Current Opinion in Chemical Biology*.

[4] Lu D. P., Qiu J. Y., Jiang B. (2002). Tetra-arsenic tetra-sulfide for the treatment of acute promyelocytic leukemia: a pilot report. *Blood*.

[5] Pharmacopoeia Commission C. (2020). *Pharmacopoeia of the People’s Republic of China Part I*.

[6] https://www.atsdr.cdc.gov/spl/resources.

[7] International Agency for Research on Cancer IARC monographs on the identification of carcinogenic hazards to humans. https://monographs.iarc.fr/agents-classified-by-the-iarc.

[8] Zhou J., Ma H., Wu Y. (2019). Lipidomic profiling of subchronic As_4_S_4_ exposure identifies inflammatory mediators as sensitive biomarkers in rats. *Metallomics*.

[9] Wu J., Shao Y., Liu J., Chen G., Ho P. C. (2011). The medicinal use of realgar (As_4_S_4_) and its recent development as an anticancer agent. *Journal of Ethnopharmacology*.

[10] Jayawardene I., Saper R., Lupoli N., Sehgal A., Wright R. O., Amarasiriwardena C. (2010). Determination of *in vitro* bioaccessibility of Pb, As, Cd and Hg in selected traditional Indian medicines. *Journal of Analytical Atomic Spectrometry*.

[11] Wu X., Wu S., Liu Y. (2018). Health risk assessment of arsenic in Realgar and NiuHuangJieDu Tablets based on pharmacokinetic study. *Journal of Trace Elements in Medicine and Biology*.

[12] Zhou L., Wang S., Hao Q. X. (2018). Bioaccessibility and risk assessment of heavy metals, and analysis of arsenic speciation Cordyceps sinensi. *Chinese Medicine*.

[13] García-Sevillano M. A., García-Barrera T., Navarro F., Gómez-Ariza J. L. (2013). Analysis of the biological response of mouse liver (*Mus musculus*) exposed to As_2_O_3_ based on integrated -*omics* approaches. *Metallomics*.

[14] Zhang M. H., Chen J. Q., Guo H. M. (2017). Combination of LC/MS and GC/MS based metabolomics to study the hepatotoxic effect of realgar nanoparticles in rats. *Chinese Journal of Natural Medicines*.

[15] Huo T., Fang Y., Zhao L. (2016). ^1^HNMR-based metabonomic study of sub-chronic hepatotoxicity induced by realgar. *Journal of Ethnopharmacology*.

[16] Huang Y., Tian Y., Li G. (2013). Discovery of safety biomarkers for realgar in rat urine using UFLC-IT-TOF/MS and ^1^H NMR based metabolomics. *Analytical and Bioanalytical Chemistry*.

[17] Wei L., Liao P., Wu H. (2009). Metabolic profiling studies on the toxicological effects of realgar in rats by ^1^H NMR spectroscopy. *Toxicology and Applied Pharmacology*.

[18] Wu X., Guan R., Liu Y., Wu S., Song M., Hang T. (2020). Comparative health risk assessment of realgar and NiuHuangJieDu tablets based on tissue arsenic levels after multiple oral administration to rats. *Journal of Ethnopharmacology*.

[19] Tinggi U., Sadler R., Ng J., Noller B., Seawright A. (2016). Bioavailability study of arsenic and mercury in traditional Chinese medicines (TCM) using an animal model after a single dose exposure. *Regulatory Toxicology and Pharmacology*.

[20] Xu W., Wang H., Chen G. (2014). A metabolic profiling analysis of the acute toxicological effects of the realgar (As_2_S_2_) combined with other herbs in Niuhuang Jiedu Tablet using ^1^H NMR spectroscopy. *Journal of Ethnopharmacology*.

[21] Xu W., Wang H., Chen G., Li W., Xiang R., Pei Y. (2013). ^1^H NMR-based metabonomics study on the toxicity alleviation effect of other traditional Chinese medicines in Niuhuang Jiedu tablet to realgar (As_2_S_2_). *Journal of Ethnopharmacology*.

[22] Lemoine L., Thijssen E., Noben J. P., Adriaensens P., Carleer R., Speeten K. V. d. (2018). A validated inductively coupled plasma mass spectrometry (ICP-MS) method for the quantification of total platinum content in plasma, plasma ultrafiltrate, urine and peritoneal fluid. *Journal of Pharmaceutical and Biomedical Analysis*.

[23] Klencsár B., Bolea-Fernandez E., Flórez M. R. (2016). Determination of the total drug-related chlorine and bromine contents in human blood plasma using high performance liquid chromatography-tandem ICP-mass spectrometry (HPLC-ICP-MS/MS). *Journal of Pharmaceutical and Biomedical Analysis*.

[24] Zhao D., Zhang Y., Wang Y. (2014). Pharmacokinetics study of hemin in rats by applying ^58^Fe-extrinsically labeling techniques in combination with ICP-MS method. *Journal of Pharmaceutical and Biomedical Analysis*.

[25] Lewchalermvong K., Rangkadilok N., Nookabkaew S., Suriyo T., Satayavivad J. (2018). Arsenic speciation and accumulation in selected organs after oral administration of rice extracts in Wistar rats. *Journal of Agricultural and Food Chemistry*.

[26] Yu H., Wu B., Zhang X. X. (2016). Arsenic metabolism and toxicity influenced by Ferric iron in simulated gastrointestinal tract and the roles of gut microbiota. *Environmental Science and Technology*.

[27] Jin P., Liang X., Xia L. (2016). Determination of 20 trace elements and arsenic species for a realgar-containing traditional Chinese medicine Niuhuang Jiedu tablets by direct inductively coupled plasma-mass spectrometry and high performance liquid chromatography-inductively coupled plasma-mass spectrometry. *Journal of Trace Elements in Medicine and Biology*.

[28] Luo J., Han X., Dou X., Zhang L., Yang S., Yang M. (2017). Accumulation of arsenic speciation and *in vivo* toxicity following oral administration of a Chinese patent medicine Xiao-Er-Zhi-Bao-Wan in rats. *Frontiers in Pharmacology*.

[29] Han X., Luo J., Zhou W., Yang S., Yang M. (2016). Determination and pharmacokinetic properties of arsenic speciation in Xiao-Er-Zhi-Bao-Wan by high-performance liquid chromatography with inductively coupled plasma mass spectrometry. *Journal of Separation Science*.

[30] Xu W., Zhang S., Jiang W., Xu S., Jin P. (2020). Arsenic accumulation of realgar altered by disruption of gut microbiota in mice. *Evidence-based Complementary and Alternative Medicine: eCAM*.

[31] Sun B., Wang X., Cao R. (2016). NMR-based metabonomics study on the effect of Gancao in the attenuation of toxicity in rats induced by Fuzi. *Journal of Ethnopharmacology*.

[32] Zhao X. J., Hao F., Huang C. (2012). Systems responses of rats to mequindox revealed by metabolic and transcriptomic profiling. *Journal of Proteome Research*.

[33] Xue L. M., Zhang Q. Y., Han P. (2014). Hepatotoxic constituents and toxicological mechanism of *Xanthium strumarium* L. fruits. *Journal of Ethnopharmacology*.

[34] Zheng X. F., Tian J. S., Liu P., Xing J., Qin X. M. (2014). Analysis of the restorative effect of *Bu-Zhong-yi-qi-tang* in the spleen-qi deficiency rat model using ^1^H-NMR-based metabonomics. *Journal of Ethnopharmacology*.

[35] Miao Y. J., Shi Y. Y., Li F. Q. (2016). Metabolomics study on the toxicity of *Annona squamosa* by ultraperformance liquid-chromatography high-definition mass spectrometry coupled with pattern recognition approach and metabolic pathways analysis. *Journal of Ethnopharmacology*.

[36] Xu H. H., Ma Z. C., Shi Q. L. (2018). Synergistic effect and different toxicities of adjuvant components of *Realgar-Indigo Naturalis* formula. *Chinese Herbal Medicines*.

[37] Zhang Q. L., Wu Q., Xie Y. Y. (2011). Tissue distribution of arsenic of Liushen pills and realgar. *Acta Pharmaceutica Sinica*.

[38] Xu W., Xu S., Zhang S., Wu X., Jin P. (2019). Arsenic bioaccessibility of realgar influenced by the other traditional Chinese medicines in Niuhuang Jiedu tablet and the roles of gut microbiota. *Evidence-based Complementary and Alternative Medicine*.

[39] Dong J., Chen H., Wu J., Wang M. Y., Zhan Z. (2011). Determination of soluble arsenic in Niuhuang Jiedu Tablets and its disassembled presciption by AFS. *Chinese Journal of Experimental Traditional Medical Formulae*.

[40] Xu Z., Yu S. S., Huang K. L. (2009). Influence of the different components in Niuhuang Jiedu Tablets on the solubility of arsenic in realgar. *Chinese Journal of Biochemical Pharmaceutics*.

