# Supplementary material for: Compatibility of Niuhuang Jiedu Tablets Results in Attenuated Arsenic Bioaccumulation and Consequent Protection against Realgar-Induced Toxicity in Mice
Source: Evid Based Complement Alternat Med. 2022 Jun 23;2022:7406694. doi: 10.1155/2022/7406694 (PMC9273386; doi:10.1155/2022/7406694)
Supplement: Supplementary Materials — Figure S1. Photomicrographs of representative sections of the liver from the control group (a), group R (b), group NJT (c), group RBC (d), group RBS (e), group RGF (f), group RRR (g), group RSR (h), group RPR (i), group RGR (j), and group RFH (k). Figure S2. Photomicrographs of representative sections of the kidney from the control group (a), group R (b), group NJT (c), group RBC (d), group RBS (e), group RGF (f), group RRR (g), group RSR (h), group RPR (i), group RGR (j), and group RFH (k). [file 7406694.f1.pdf]

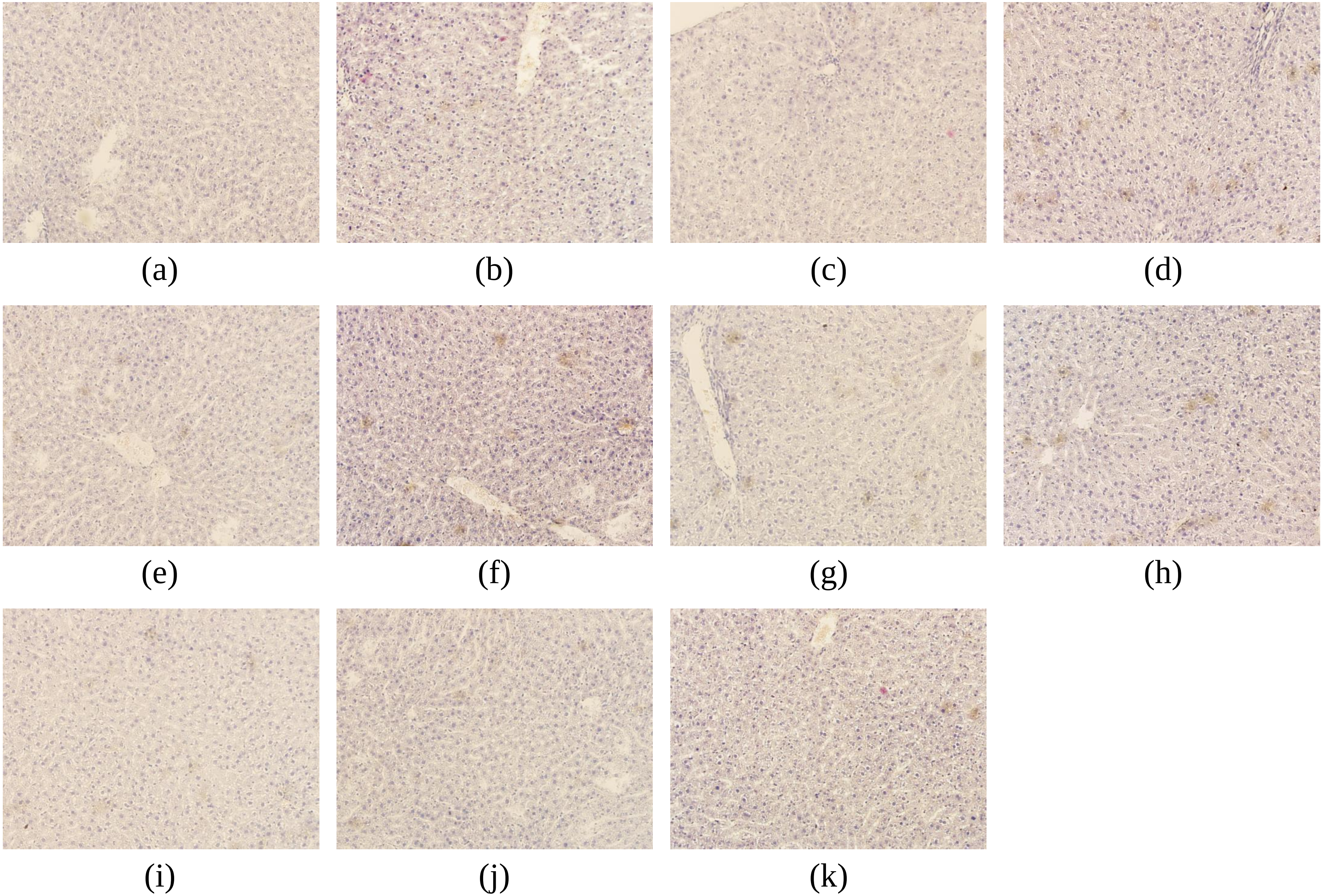

Figure S1. Photomicrographs of representative sections of livers from the control group (a), group R (b), group NJT (c), group RBC (d), group RBS (e), group RGF (f), group RRR (g), group RSR (h), group RPR (i), group RGR (j), group RFH (k).

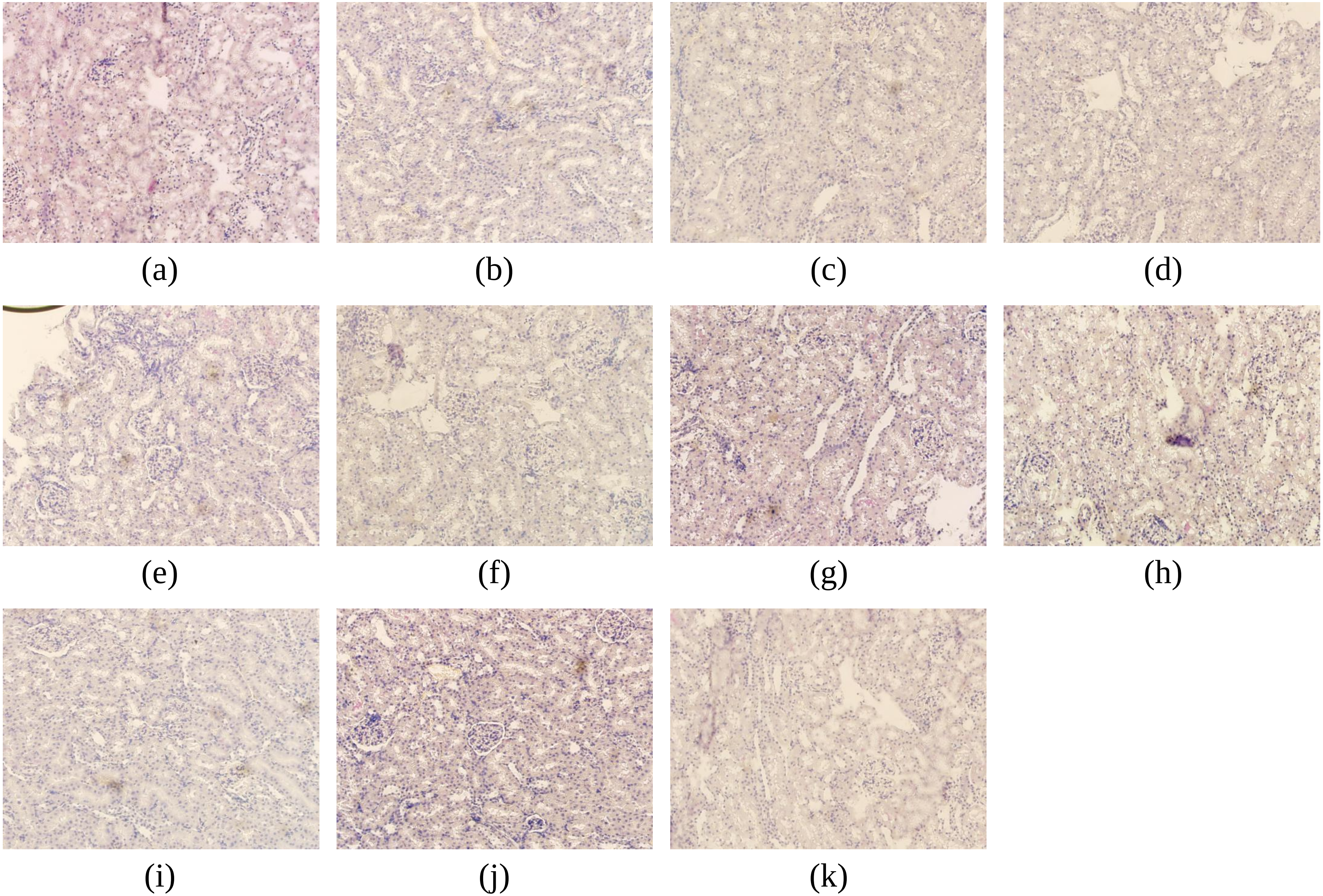

Figure S2. Photomicrographs of representative sections of kidneys from the control group (a), group R (b), group NJT (c), group RBC (d), group RBS (e), group RGF (f), group RRR (g), group RSR (h), group RPR (i), group RGR (j), group RFH (k).
